# LncRNA SNAI3-AS1 promotes PEG10-mediated proliferation and metastasis via decoying of miR-27a-3p and miR-34a-5p in hepatocellular carcinoma

**DOI:** 10.1038/s41419-020-02840-z

**Published:** 2020-08-11

**Authors:** Yarui Li, Dan Guo, Guifang Lu, Abu Taiub Mohammed Mohiuddin Chowdhury, Dan Zhang, Mudan Ren, Yifei Chen, Ruhua Wang, Shuixiang He

**Affiliations:** Department of Gastroenterology, The First Affiliated Hospital of Xi’an Jiaotong University, 710061 Xi’an, Shaanxi P. R. China

**Keywords:** Metastasis, Oncogenes

## Abstract

During recent years, long noncoding RNAs (lncRNAs) have received focal attention due to their important function in cancer regulation. Though the relation between lncRNA SNAI3-AS1 and the development of hepatocellular carcinoma (HCC) has been described in our previous study, the role and the exact mechanism of SNAI3-AS1 are still unclear. In this study, qRT-PCR analysis revealed that the expression of SNAI3-AS1 was elevated and was correlated with the levels of PEG10 in HCC tissues. Through functional experiments, we determined that knockdown of SNAI3-AS1 and PEG10 inhibited the proliferation and metastasis, whereas overexpression of SNAI3-AS1 and PEG10 promoted the proliferation and metastasis of HCC cells. In addition, rescue experiments confirmed that upregulation of PEG10 partially restored cell function inhibition induced by SNAI3-AS1 knockdown. Therefore, we hypothesized that PEG10 may be regulated by SNAI3-AS1, which in turn mediates the malignant biological processes of HCC cells regulated by PEG10. Further bioinformatics analysis and mechanistic experiments showed that SNAI3-AS1 functions as a competing endogenous RNA (ceRNA) to activate PEG10 by acting as a sponge for miR-27-3p and miR-34a-5p. In summary, our study revealed that SNAI3-AS1 is a tumor regulator of PEG10 in the progression of HCC, and may contribute to the improvement of HCC diagnosis and therapy.

## Introduction

Hepatocellular carcinoma (HCC) is the principal cause of mortality among cirrhotic patients and the third cause of cancer-related death worldwide^1,2^. Liver cancer is divided into cholangiocarcinoma, HCC, and mixed HCC according to different cell origin, and among them, HCC is the most common type. The character of fast growth and unobvious symptoms in the early stage takes the main charge for the unfavorable diagnosis and prognosis of HCC. Hepatocarcinogenesis is often described as a complex step involving multiple genes and genetic alterations that ultimately lead to malignant transformation of hepatocytes^3–5^. Despite significant advances in diagnosis and management, the molecular biology of HCC remains poorly understood. Therefore, looking for crucial tumor-related molecular and identifying the underlying mechanisms in HCC are urgently needed for molecular diagnostics and targeted therapies.

Long noncoding RNAs (lncRNAs) are a type of transcribed RNA molecules and with length of more than 200 nucleotides, which play a crucial role in cancer progression^6,7^. LncRNAs are responsible for a number of biological processes that involve the process of tumorigenesis and metastasis by the epigenetic, transcriptional, and post-transcriptional mechanisms^8,9^. The competing endogenous RNA (ceRNA) is the mostly recognized working mechanism of lncRNAs. CeRNA theory hypothesizes that RNA transcriptions include lncRNA communication through a new “language” mediated by microRNA response elements (MREs)^10^. Several lncRNAs had been identified to take part in the pathogenesis of HCC as oncogenes or tumor-suppressor genes through competitive binding to miRNA^11,12^. For example, our previous study demonstrated that lncRNA SNHG5 functions as an oncogene and promotes cell growth and metastasis by binding miR-26a-5p^13^. Although multiple lncRNAs have been identified as a regulator in HCC pathogenesis, the relationship between the majority of lncRNAs and HCC is yet to be explored.

SNAI3-AS1 has been identified as an lncRNA that located within the amplified 16q24 locus. Our previous study detected that SNAI3-AS1 might act as a tumor oncogene in HCC^14^. In order to further explore the molecular mechanism of SNAI3-AS1 in HCC, we screened SNAI3-AS1-related ceRNA molecules. Paternally expressed gene 10 (PEG10), first identified as an imprinted gene, was a potential biomarker in the progressive development of HCC^15^. Simultaneously, bioinformatics analysis showed that PEG10 was the target gene of miRNAs. In the current study, we focused on the association of the lncRNA SNAI3-AS1 and PEG10 in HCC tumorigenesis. Besides, the role of SNAI3-AS1 and PEG10 in HCC disease progression and outcome was supported by the clinical, pathological, and expression data that were obtained from the HCC patients. These findings have exposed a novel regulatory mechanism of SNAI3-AS1 in HCC.

## Materials and methods

### Patients and tissue samples

Forty-six cases of HCC tissues and matched para-tumor tissues (3–5 cm distal to the edge of tumor) used in this study were collected under the permission of primary HCC patients during tumorectomy in the First Affiliated Hospital of Xi’an Jiaotong University. Hospital ethical committee approval and informed written consents were obtained in all cases. The diagnosis was confirmed by histopathology. The prognostic analysis data were obtained from the KM plotter (http://kmplot.com/analysis/index.php?p=background) database.

### Cell culture

The human HCC cell lines and the immortalized hepatic cells (Human) LO2 were obtained from the Chinese Academy of Sciences Cell Bank (Shanghai, China). The cells were cultured in the Dulbecco’s modified Eagle’s medium *(*DMEM)/high glucose (Hyclone, USA) in a humidified incubator at 37 °C temperature and 5% CO_2_ concentration. Ten percent FBS (fetal bovine serum; Gibco, USA) and penicillin–streptomycin (100 U/mL and 100 μg/mL, respectively) were added in the DMEM/high-glucose medium prior to culture.

### Plasmids and cell transfection

The siRNA (small-interfering RNA) against SNAI3-AS1 and PEG10, SNAI3-AS1 and PEG10-overexpression plasmids and the SNAI3-AS1-knockdown plasmids (SNAI3-AS1 shRNA with a corresponding negative control shRNA—NC shRNA) were designed by GenePharma (Shanghai, China). MiR-27a-3p mimics, miR-34a-5p mimics, NC mimic, miR-27a-3p inhibitor, miR-34a-5p inhibitor, and NC inhibitor were purchased from RiboBio (Guangzhou, China). Lipofectamine 2000 (Invitrogen, Carlsbad, CA, USA) was used for cell transfection by the manufacturer’s protocol. For a stable cell transfection, the transfected cells were selected by 5 μg/mL puromycin-containing medium. The puromycin-resistant cell clones were established after 2 weeks. Quantitative real-time PCR (qRT-PCR) analysis was applied to detect gene expression level to value the silenced or overexpression efficiency.

### RNA isolation and qRT-PCR

Trizol reagent (Invitrogen, Carlsbad, CA, USA) was used to extract the total RNA from the cultured cells and the collected HCC tissues. The qRT-PCR was carried out as previously described^13^. The relative expression of genes was calculated using the 2^−ΔΔCt^ method.

Primers sequences for PCR were as follows:

SNAI3-AS1—F 5′-GCGTTATGTCGTTTGGTTGATG-3′

SNAI3-AS1—R 5′-TGGCAGGAATGAGGTGAGC-3′

β-actin—F 5′-ATCGTGCGTGACATTAAGGAGAAG-3′

β-actin—R 5′AGGAAGGAAGGCTGGAAGAGTG-3′

PEG10—F 5′-GGACCTGGATTGGAACGAG-3′

PEG10—R 5′-GAGCAGACAGCGACTTGG-3′.

### Cell-proliferation assays

The MTT (3-[4,5-dimethylthiazol-2-yl]-2,5-diphenyltetrazolium bromide) assay was executed as previously reported^13^. For EdU incorporation assay, HCC cells were seeded in 96-well plates (2 × 10^3^) supplemented with complete growth medium and followed by different transfection 24 h later, and then the procedure was carried out according to the manufacturer’s protocol with EdU detection kits (Keygen, Nanjing, China). Triplicate experiments were performed for each assay.

### Cell migration and invasion assays

#### Transwell assay

After cell transfection, 5 × 10^4^ cells in serum-free medium were seeded on uncoated (for migration assays) and Matrigel-coated (for invasion assays; BD Bioscience, USA) upper chambers (Merck Millipore), respectively. Culture medium containing 10% FBS was supplemented into the lower wells and incubated for further 24 h, followed by wiping off the noninvaded or nonmigrated cells. Then the filters were fixed in 90% ethanol for 10 min and stained by crystal violet for 15 min. Five random fields were counted per chamber by using an inverted microscope (Leica, Germany).

#### Wound-healing assay

After transfection, the HCC cells were cultured for 24 h to achieve 90% cell density in six-well plates. A 10-µl sterile tip was used to scratch the culture plate and the floating cells were washed off. The wound size was measured and photographed at 0, 24, and 48 h.

### Western blot analysis

The total protein from the cultured HCC cells and the tissue samples was isolated by RIPA (Beyotime, Haimen China). Proteases and phosphatases were added to RIPA to prevent protein degradation. BCA detection Kit (Keygen, Nanjing, China) was used for qualification according to the manufacturer’s protocol. Protein samples were loaded for electrophoresis (5% gel for concentration and 10% for separation). Following electrophoresis, the proteins were transferred on a PVDF membrane (Merck Millipore) and were blocked by 5% nonfat milk for 1 h. Then the PVDF membrane was incubated overnight at 4 °C with the primary antibodies (PEG10; Abcam) at 1:1000 dilution. The next day, the membrane was incubated with secondary antibodies (a dilution rate of 1:5000; Zhuangzhi Biology, China) for 1 h at room temperature. The protein bands were evaluated by ECL immunoblotting kit following the manufacturer’s protocol (Millipore, USA).

### Luciferase reporter assay

Cells were placed in 96-well plates and cultured to 60–80% confluence before transfection. Wild and mutant reporter plasmids of SNAI3-AS1 (wt-SNAI3-AS1-luc, mut-SNAI3-AS1-luc) and PEG10 (wt-PEG10-luc, mut-PEG10-luc), which contain a miR-27a-5p or miR-34a-3p binding sites, were individually synthesized by GeneChem (GenePharma, Shanghai, China). The synthesized reporter plasmids were co-transfected with miR-27a-5p or miR-34a-3p mimics/inhibitor or mimic control/inhibitor control, respectively. Lipofectamine 2000 reagent (Invitrogen, Carlsbad, CA) was used for transfection. The alteration of the luciferase activity was devaluated in each group by Dual Luciferase Assay Kit according to the manufacturer’s protocol. Renilla luciferase activity was used as a control.

### In vivo nude mouse models

Four-week aged nude mice (male) were purchased from the “Animal Care and Use Committee of Dalian Medical University Ltd”. All the mice were kept under sterile specific-pathogen-free (SPF) environment. 1 × 10^6^ cells with stable knockdown of SNAI3-AS1 and with the corresponding negative control were injected subcutaneously or intravenously for the determination of tumor growth and metastasis. The subcutaneous formatted tumor nodes (32 days) and the lungs from the metastatic group (60 days) were harvested for further detection. The study was carried out according to the Guide line for the “Care and Use of Laboratory Animals of the National Institutes of Health” and was approved by the Medical Ethics Committee of the Experimental Animal Center of Xi’an Jiaotong University.

### Statistical analysis

SPSS 23.0 (IBM, SPSS, Chicago, IL, USA) and GraphPad Prism V7.0 (GraphPad Software, CA, USA) were used to analyze the results. One-way ANOVA or Student’s *t*-test was done to evaluate the difference between the groups. The Kaplan–Meier test was used to assess survival curves. The log-rank test was used to determine the statistical differences between survival curves. Correlation between two groups was analyzed using Pearson’s correlation coefficient analysis. A two-tailed *P* < 0.05 was considered as statistically significant, and *P* < 0.01 was very significant.

## Results

### SNAI3-AS1 and PEG10 was upregulated in HCC tissues and correlated with poor prognosis in HCC patients

**Fig. 1:**
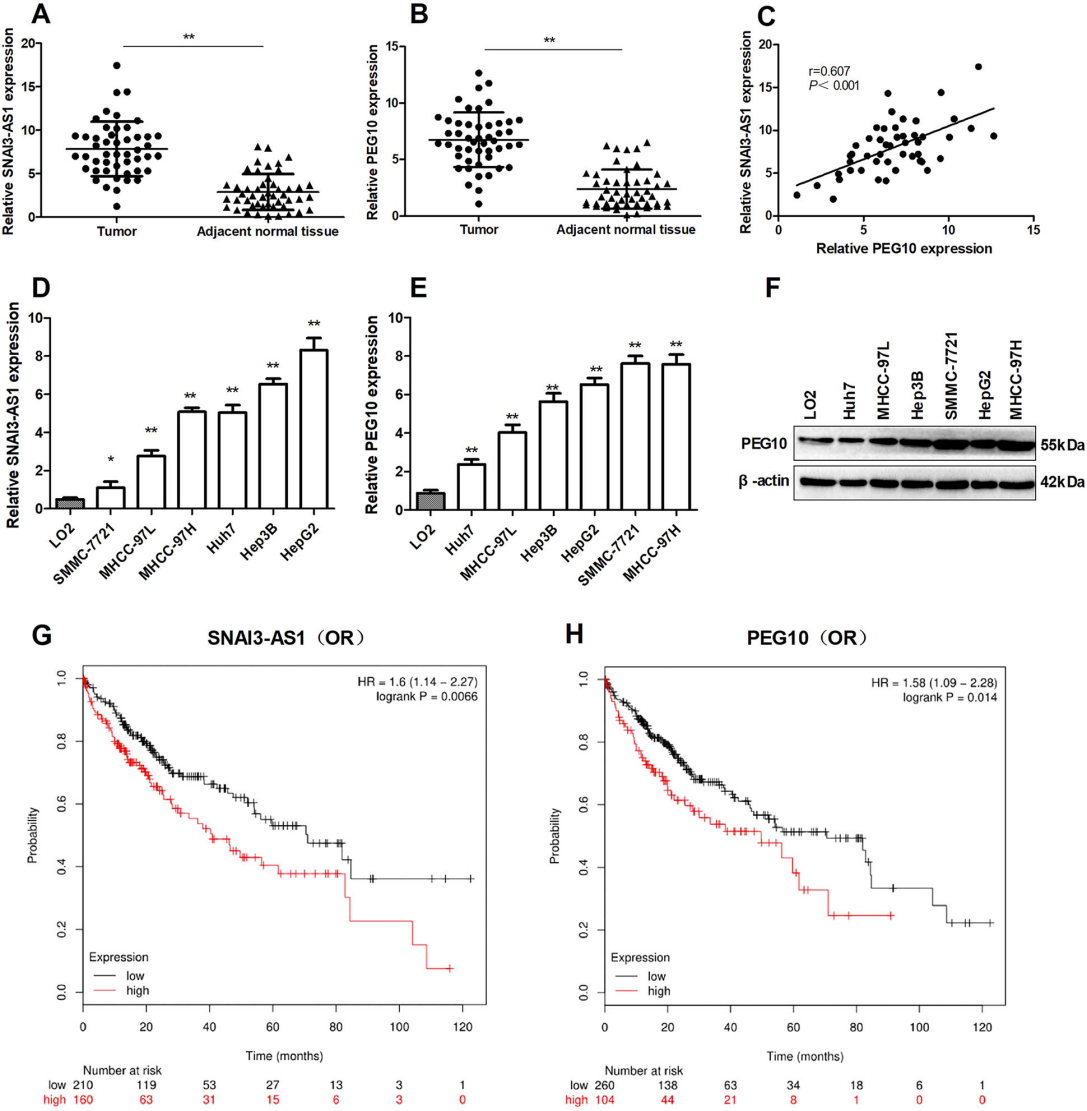
High SNAI3-AS1 and PEG10 expression predicts poor HCC patients’survival. QRT-PCR analysis of SNAI3-AS1 (**a**) and PEG10 (**b**) expression in 46 patients with HCC and matched adjacent normal tissues. **P < 0.01. **c** The positive relationship between SNAI3-AS1 and PEG10 in HCC tissues. QRT-PCR analysis of SNAI3-AS1 (**d**) and PEG10 (**e**) expression in HCC cell lines and the immortalized human hepatic cell line LO2, *P < 0.05; **P < 0.01. **f** Western blot analysis of PEG10 expression in HCC cell lines and LO2. **g**, **h** Survival plots (Kaplan–Meier) based on SNAI3-AS1 and PEG10 expression levels in HCC patients. The median level of gene expression is used as the cutoff.

We examined SNAI3-AS1 and PEG10 expression by qRT-PCR in 46 pairs of HCC and matched adjacent normal tissues. According to the qRT-PCR results, SNAI3-AS1 and PEG10 were overexpressed in HCC tissues compared with matched adjacent normal tissues (Fig. 1a, b). The data analysis has revealed a positive relationship between the expression of SNAI3-AS1 and PEG10 in HCC tissues (Fig. 1c). Further, SNAI3-AS1 and PEG10 were also found to be upregulated in HCC cell lines compared with immortalized, normal human hepatic cell line LO2 (Fig. 1d, e). Besides, western blotting verified that PEG10 protein was upregulated in HCC cell lines (Fig. 1f). To analyze the relationship of SNAI3-AS1 and PEG10 expression about HCC patient’s prognosis, the Kaplan–Meier and log-rank analyses (http://kmplot.com/analysis/) were used to predict the overall survival (OS) of HCC patients. The results showed that patients with higher SNAI3-AS1 and PEG10 expression had shorter OS than patients with low levels of SNAI3-AS1 and PEG10 (Fig. 1g, h). These data indicated that overexpressed SNAI3-AS1 and PEG10 were associated with the development and progression of HCC.

### SNAI3-AS1 and PEG10 regulate cell proliferation, migration, and invasion of HCC cells

**Fig. 2:**
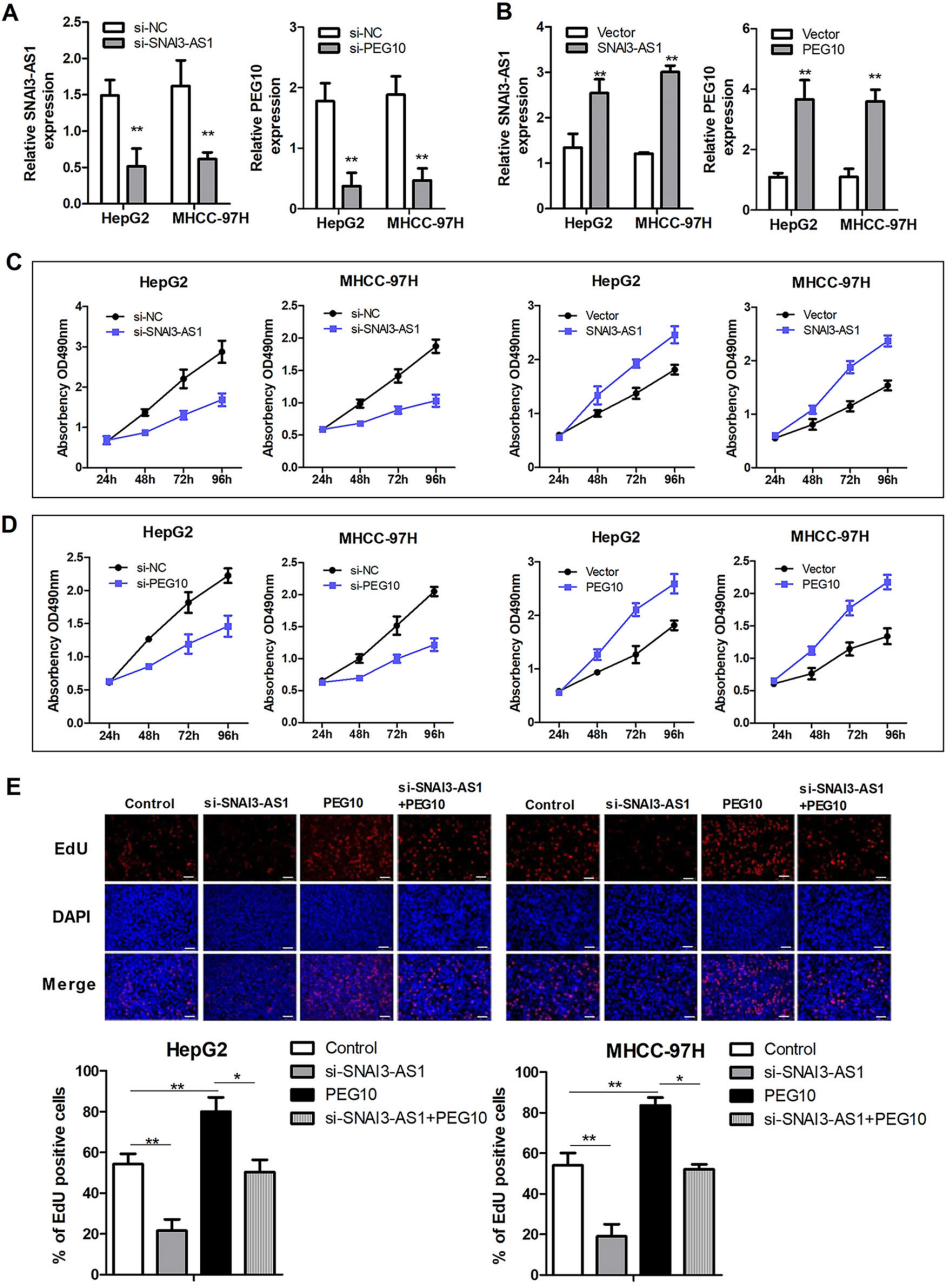
SNAI3-AS1 and PEG10 regulate the proliferation of HCC cells. **a** The expression of SNAI3-AS1 and PEG10 was downregulated in the siRNA group. **b** The expression of SNAI3-AS1 and PEG10 was upregulated in the plasmid-transfection group. MTT assays (**c**, **d**) and EdU assay (**e**) showed that silencing of SNAI3-AS1 and PEG10 inhibited the proliferation, while upregulation of SNAI3-AS1 and PEG10 promoted the proliferation of HCC cells.*P < 0.05; **P < 0.01. Scale bars: 20 μm.

To verify the effects of SNAI3-AS1 and PEG10 on the biological function of HCC cells in vitro, the siRNAs were synthesized for knocking down the expression of SNAI3-AS1 and PEG10 (Fig. 2a), and SNAI3-AS1- and PEG10-overexpression plasmids were used for upregulation in HepG2 and MHCC-97H cell lines (Fig. 2b). We investigated the role of SNAI3-AS1 and PEG10 in proliferation using MTT and Edu assays. The MTT assay showed that inhibition of SNAI3-AS1 and PEG10 reduced cell proliferation, while the overexpression of SNAI3-AS1 and PEG10 promoted cell proliferation (Fig. 2c, d). The result of the Edu assay (Fig. 2e) showed similar results with respect to proliferation; in addition, the overexpression plasmid of PEG10 can partially restore the inhibitory effect of siRNA-SNAI3-AS1 on the proliferation of HCC cells. These data suggested that SNAI3-AS1 and PEG10 promote the proliferation of HCC cells.

### SNAI3-AS1 and PEG10 regulate cell migration and invasion ability of HCC cells

**Fig. 3:**
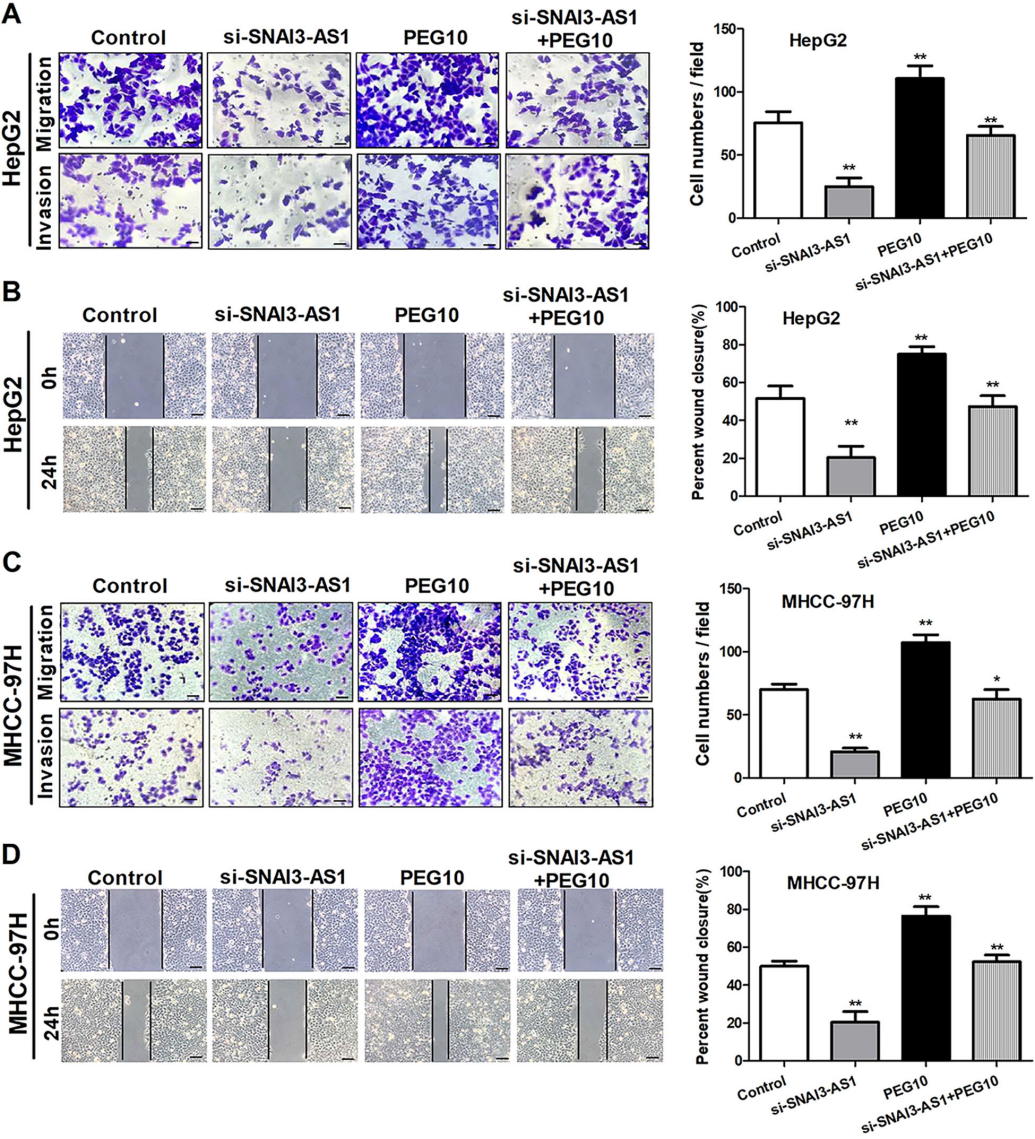
SNAI3-AS1 and PEG10 regulate cell migration and invasion of HCC cells. **a, c** Transwell assay and **b**, **d** wound-healing assay showed that downregulation of SNAI3-AS1 inhibited cell migration and invasion, while the opposite result was observed in PEG10 upregulation. Meanwhile, the ability of migration and invasion regulated by siRNA-SNAI3-AS1 could be partially rescued by PEG10 upregulation. *P < 0.05; **P < 0.01. Scale bars: 20 μm.

The cell migration and the invasion ability of the HCC cells were evaluated by Transwell and wound-healing assays. The results showed that overexpression of PEG10 significantly promoted the migration and invasion abilities of HCC cells, while the opposite phenomena were observed when SNAI3-AS1 was knocked down (Fig. 3a, c). The wound-healing assay was used to assess the effect of SNAI3-AS1 and PEG10 on tumor cell mobility. After 24 h of migration, inhibition of SNAI3-AS1 suppressed cell migration, and upregulation of PEG10 promoted cell migration (Fig. 3b, d). Moreover, the ability of migration and invasion regulated by siRNA-SNAI3-AS1 could be partially rescued by PEG10 upregulation. These data suggested that SNAI3-AS1 and PEG10 are able to promote the migration and invasion of HCC cells.

### SNAI3-AS1 promotes HCC cell proliferation and migration in vivo

**Fig. 4:**
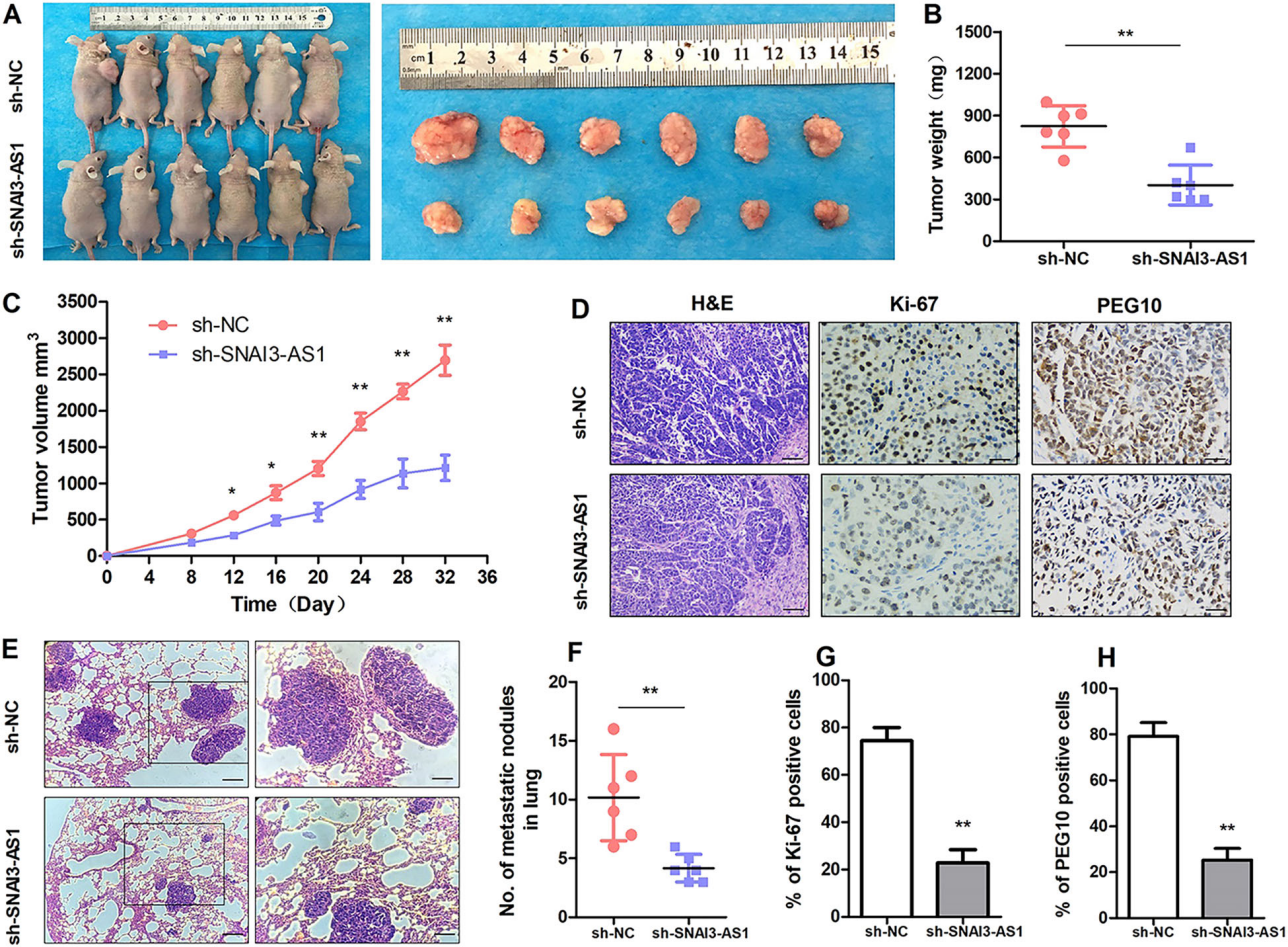
Knockdown of SNAI3-AS1 represses tumor growth and metastasis in vivo. **a** Representative images of nude mice models and formed tumors that were subcutaneously injected with SNAI3-S1-shRNA and NC cells. **b, c** Effect of SNAI3-S1 knockdown on HCC growth in vivo according to the tumor weight and tumor growth curve. *P < 0.05, **P < 0.01. **d**, **g**, **h** H&E and immunofluorescence staining using antibodies against Ki-67and PEG10 was used to assess proliferation capacity.**P < 0.01. Scale bars: 20 μm. **e**, **f** Representative images of pulmonary metastatic models and H&E staining of metastatic nodules in the lungs (×200 and ×400), **P < 0.01. Scale bars: 20 and 10 μm.

We investigated the in vivo activity of SNAI3-AS1 in nude mice. To confirm whether SNAI3-AS1 affects tumorigenesis, HepG2 cells stably transfected with SNAI3-S1-shRNA and NC were subcutaneously injected into male nude mice. After 4 weeks, we found that SNAI3-S1-shRNA dramatically inhibited tumor growth compared with the NC group (Fig. 4a), and tumor weight (Fig. 4b) and volume (Fig. 4c) were dramatically decreased in the SNAI3-S1-shRNA group. In addition, an immunostaining analysis of xenografted tumor tissues revealed that SNAI3-S1 inhibition dramatically reduced Ki-67 expression, while it increased PEG10 expression compared with the NC group (Fig. 4d). Next, we evaluated the effects of SNAI3-S1 on HCC tumor metastasis using the pulmonary nude mouse model. Hematoxylin and eosin staining of lung sections and statistics showed that SNAI3-S1 knockdown reduced the number and size of visible lung metastases (Fig. 4e, f). These results demonstrated that SNAI3-AS1 promotes HCC cell proliferation and migration in vivo.

### SNAI3-AS1 regulated PEG10 expression through the ceRNA sponging of miR-27a-3p and miR-34a-5p

**Fig. 5:**
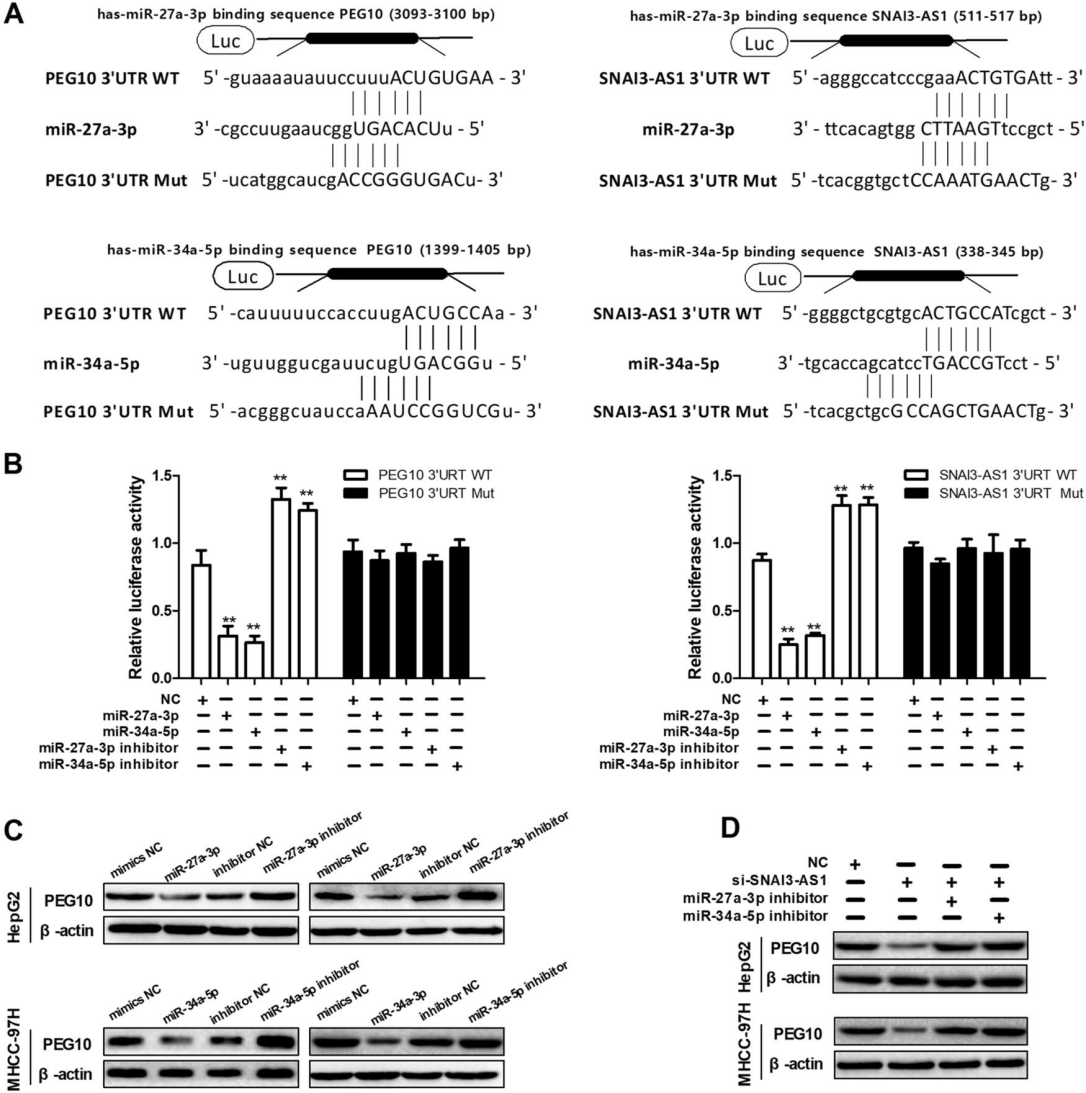
SNAI3-AS1 regulated PEG10 expression through the ceRNA sponging of miR-27a-3p and miR-34a-5p. **a** The binding sites between SNAI3-AS1 or PEG10 and miR-27a-3p or miR-34a-5p, data from starbase V3.0 and TargetScan. **b** Luciferase reporter assay was applied to verify the targeted binding effect between SNAI3-AS1 or PEG10 and miRNAs. *P < 0.05, **P < 0.01. **c** The PEG10 protein levels after miRNA overexpression or downregulation were detected by western blot. **d** Western blot analyses of PEG10 expression after knockdown of SNAI3-AS1, while the inhibition of miRNAs reversed the change in PEG10 expression.

To further investigate the mechanism underlying the role of SNAI3-AS1 and PEG10 in HCC tumorigenesis, we used two database, starbase V3.0 (ref. ^16^) and TargetScan^17^ for biological information prediction, and found that both SNAI3-AS1 and PEG10 are potential targets of miR-27a-3p and miR-34a-5p (Fig. 5a). Next, the dual-luciferase reporter assay was used to test whether SNAI3-AS1 and PEG10 are targets of miRNAs. The luciferase assays showed that the luciferase activity was reduced in HEK293T cells that were co-transfected with miR-27a-3p or miR-34a-5p and SNAI3-AS1-WT or PEG10-WT; in addition, the luciferase activity was increased in HEK293T cells when co-transfected with miR-27a-3p inhibitor or miR-34a-5p inhibitor and SNAI3-AS1-WT or PEG10-WT. However, mutating these putative binding sites of miR-27a-3p and miR-34a-5p (SNAI3-AS1-Mut or PEG10-Mut) resulted in compete abrogation of the above effects (Fig. 5b). These results confirmed that miR-27a-3p and miR-34a-5p target SNAI3-AS1 and PEG10. Furthermore, western blot analysis showed that the upregulation of miR-27a-3p and miR-34a-5p in HepG2 and MHCC-97H cells triggered a significant decrease in PEG10 protein expression, while downregulation of miR-27a-3p and miR-34a-5p increased PEG10 expression in HCC cells (Fig. 5c). As a rescue experiment, inhibition of miR-27a-3p and miR-34a-5p expression in SNAI3-AS1-knockdown cells reversed the decrease in PEG10 expression (Fig. 5d). Taken together, we provided evidence that SNAI3-AS1 acts as an endogenous “sponge” by binding miR-27a-3p and miR-34a-5p and thus abolishing miRNA-induced repression of PEG10.

### SNAI3-AS1 promotes HCC cell proliferation and metastasis through miR-27a-3p and miR-34a-5p

**Fig. 6:**
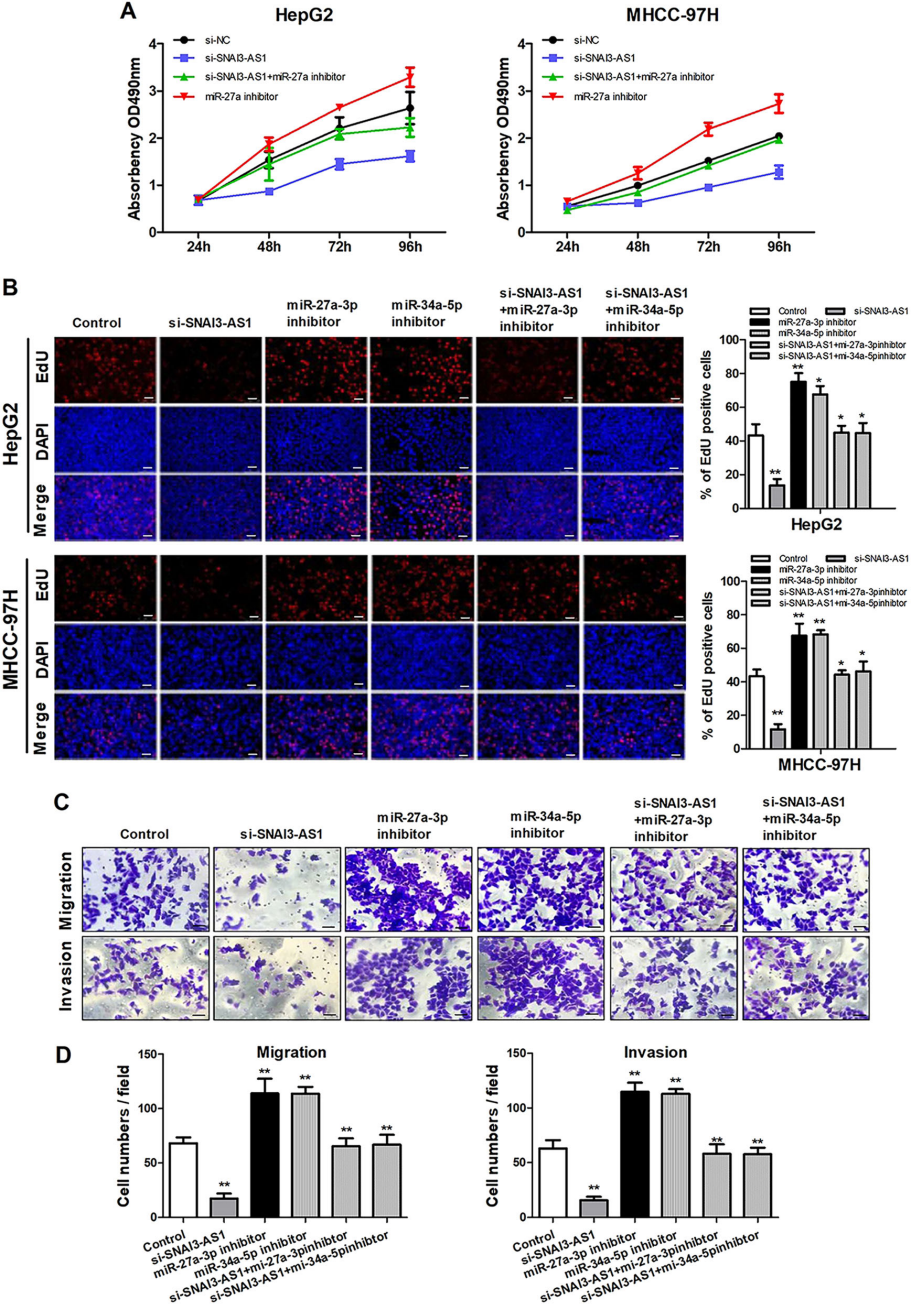
The function of SNAI3-AS1 is partially dependent on miR-27a-3p and miR-34a-5p inhibition. MTT assays (**a**) and EdU assay (**b**) were used to determine SNAI3-AS1-siRNA HCC cell growth in response to miR-27a-3p and miR-34a-5p inhibitor. *P < 0.05; **P < 0.01. Transwell assay (**c, d**) was used to determine SNAI3-AS1-siRNA HCC cell migration and invasion in response to miR-27a-3p and miR-34a-5p inhibitor. *P < 0.05; **P < 0.01. Scale bars: 20 μm.

**Fig. 7:**
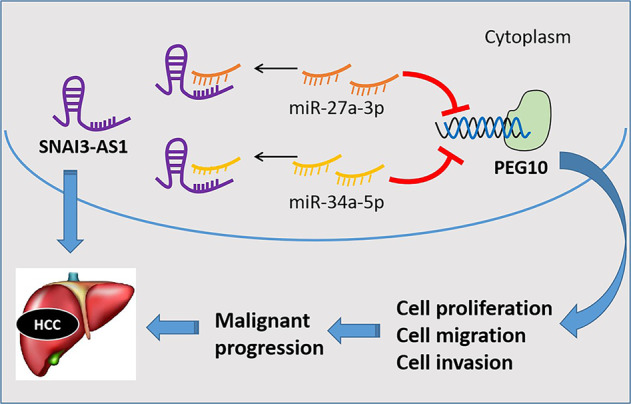
Schematic representations of pathways modulated by SNAI3-AS1 in HCC. SNAI3-AS1 is upregulated in HCC and promotes HCC progression via a ceRNA pattern by sponging miR-27a-3p and miR-34a-5p, which activate PEG10.

Since we have demonstrated that SNAI3-AS1 promoted HCC progression, and directly binds to miR-27a-3p and miR-34a-5p, we next investigated the regulation of HCC cell growth and metastasis by rescue experiments. MTT assay was used to determine SNAI3-AS1-siRNA HCC cell growth in response to miR-27a-3p and miR-34a-5p inhibitor transfection. The results showed that cell proliferation of HepG2 and MHCC-97H cells was promoted with miR-27a-3p and miR-34a-5p inhibition; however, the inhibitory effect of SNAI3-AS1-siRNA on HCC cell growth could be partially restored by miR-27a-3p and miR-34a-5p inhibition (Fig. 6a). Consistent with the MTT assay results, the EdU (Fig. 6b) and Transwell assays (Fig. 6c, d) indicated that the inhibitory effect of SNAI3-AS1-siRNA on HCC cell growth, migration, and invasion could be partially restored by miR-27a-3p and miR-34a-5p inhibition. Overall, these results suggested that the function of SNAI3-AS1 is partially dependent on miR-27a-3p and miR-34a-5p in HCC cells (Fig. 7).

## Discussion

In recent years, the discovery of a large number of noncoding RNA transcripts in the human genome has dramatically changed our understanding of complex diseases, including cancer biology. The dysregulation of lncRNA in the tumor reflects the extent of disease progression to some extent and maybe an independent risk factor for predicting the prognosis of patients^18–20^. For example, HULC, the first discovered lncRNA, was specifically highly expressed in liver cancer tissues^21^. At the same time, studies have shown that HULC is overexpressed in colon cancer liver metastases and can be detected in blood, which can be used as a molecular marker for predicting liver metastasis of colon cancer^22,23^. Other than this, a number of lncRNAs, such as HOTAIR^24^, NEAT1 (ref. ^25^), and MALAT1 (ref. ^26^), have been described to be involved in HCC disease development and progression.

In the current study, we confirmed the marked upregulation of the PEG10 and lncRNA SNAI3-AS1 in the HCC. Of note, we believe that the gene expression obtained from the fresh primary tumor samples without any prior HCC therapy increases the value of our work. In addition, our data clearly indicate that the prognosis and progression of HCC patients can be predicted by the expression level of these two genes. These findings are similar to other studies that the upregulation of lncRNAs was correlated with a poor HCC prognosis.

LncRNAs unleash their functions in a wide variety of mechanisms such as scafolding nuclear and cytoplasmic complexes, co-transcriptional regulations, gene expression modulation, etc.^27^. Among these, one factor related to lncRNAs that has received considerable attention is the ceRNA hypothesis, which demonstrated that some lncRNAs can serve as miRNA “sponges” by sharing common MREs, thus achieving regulation of downstream target genes^28–30^. These interactions influence post-transcriptional regulation by inhibiting available miRNA activity. For example, lncRNA LINC02418 can serve a value in the diagnosis of colorectal cancer by regulating MELK expression and acting as a ceRNA^31^. LncRNA HOTAIR functions as a miR-34a-5p sponge, sequestering this miRNA and thereby down regulating genes linked to apoptosis through the Notch signaling pathway^32^. In this study, we speculated that SNAI3-AS1 functions as a ceRNA by sponging miRNAs. To fully uncover the molecular mechanism, we investigated the effect of SNAI3-AS1 and PEG10 in hepatocarcinogenesis. Bioinformatics prediction combined with experimental analysis indicated the interaction between SNAI3-AS1/PEG10 and miR-27a-3p/miR-34a-5p. The luciferase assay was used to confirm that miR-27a-3p/miR-34a-5p target both SNAI3-AS1 and PEG10. Finally, our studies indicated that SNAI3-AS1 acted as a competing endogenous RNA for miR-27a-3p and miR-34a-5p, thus increasing the expression of PEG10.

In conclusion, using a combination of human-derived data and in vivo/vitro approaches, our study revealed the molecular axis comprising SNAI3-AS1 and PEG10 as a key player in HCC progression. We hope that the future studies will validate SNAI3-AS1 as a predictive biomarker in HCC progression and development. Meanwhile, the present study first demonstrated that SNAI3-AS1 could decoy two miRNAs (miR-27a-3p and miR-34a-5p) to facilitate PEG10-mediated proliferation and metastasis via a ceRNA network. This provides a novel insight into the mechanisms of hepatocarcinogenesis and a better understanding for possibly potential mechanisms of pathogenesis and molecular therapeutic strategy for HCC.

## Data Availability

The data and material in this study are available.

## References

[1] Han ZG (2012). Functional genomic studies: insights into the pathogenesis of liver cancer. Annu. Rev. Genomics Hum. Genet..

[2] El-Serag HB, Rudolph KL (2007). Hepatocellular carcinoma: epidemiology and molecular carcinogenesis. Gastroenterology.

[3] Khemlina G, Ikeda S, Kurzrock R (2017). The biology of hepatocellular carcinoma: implications for genomic and immune therapies. Mol. Cancer.

[4] Lee SM (2016). Interplay of genetic and epigenetic alterations in hepatocellular carcinoma. Epigenomics.

[5] Schlaeger C (2008). Etiology-dependent molecular mechanisms in human hepatocarcinogenesis. Hepatology.

[6] Jiang MC, Ni JJ, Cui WY, Wang BY, Zhuo W (2019). Emerging roles of lncRNA in cancer and therapeutic opportunities. Am. J. Cancer Res..

[7] Shi X, Sun M, Liu H, Yao Y, Song Y (2013). Long non-coding RNAs: a new frontier in the study of human diseases. Cancer Lett..

[8] Fatica A, Bozzoni I (2014). Long non-coding RNAs: new players in cell differentiation and development. Nat. Rev. Genet..

[9] Dykes IM, Emanueli C (2017). Transcriptional and post-transcriptional gene regulation by long non-coding RNA. Genomics Proteomics Bioinformatics.

[10] Qu J (2015). Competing endogenous RNA in cancer: a new pattern of gene expression regulation. Int. J. Clin. Exp. Med..

[11] Huang Y, Xiang B, Liu Y, Wang Y, Kan H (2018). LncRNA CDKN2B-AS1 promotes tumor growth and metastasis of human hepatocellular carcinoma by targeting let-7c-5p/NAP1L1 axis. Cancer Lett..

[12] Wang Y (2018). Long non-coding RNA DSCR8 acts as a molecular sponge for miR-485-5p to activate Wnt/beta-catenin signal pathway in hepatocellular carcinoma. Cell Death Dis..

[13] Li Y (2018). Long non-coding RNA SNHG5 promotes human hepatocellular carcinoma progression by regulating miR-26a-5p/GSK3beta signal pathway. Cell Death Dis..

[14] Li Y (2019). Long non-coding RNA SNAI3-AS1 promotes the proliferation and metastasis of hepatocellular carcinoma by regulating the UPF1/Smad7 signalling pathway. J. Cell Mol. Med..

[15] Jie X (2007). Androgen activates PEG10 to promote carcinogenesis in hepatic cancer cells. Oncogene.

[16] Li JH, Liu S, Zhou H, Qu LH, Yang JH (2014). starBase v2.0: decoding miRNA-ceRNA, miRNA-ncRNA and protein-RNA interaction networks from large-scale CLIP-Seq data. Nucleic Acids Res..

[17] Agarwal, V., Bell, G. W., Nam, J. W., Bartel, D. P. Predicting effective microRNA target sites in mammalian mRNAs. *Elife* **4**, e05005 (2015). 10.7554/eLife.05005 PMC4532895 26267216

[18] Tang J (2019). LncRNA GLCC1 promotes colorectal carcinogenesis and glucose metabolism by stabilizing c-Myc. Nat. Commun..

[19] Zhong Y (2019). Long noncoding RNAs as potential biomarkers and therapeutic targets in gallbladder cancer: a systematic review and meta-analysis. Cancer Cell Int..

[20] Quan J (2018). LncRNA as a diagnostic and prognostic biomarker in bladder cancer: a systematic review and meta-analysis. Onco Targets Ther..

[21] Panzitt K (2007). Characterization of HULC, a novel gene with striking up-regulation in hepatocellular carcinoma, as noncoding RNA. Gastroenterology.

[22] Xie H, Ma H, Zhou D (2013). Plasma HULC as a promising novel biomarker for the detection of hepatocellular carcinoma. Biomed. Res. Int..

[23] DiStefano JK (2017). Long noncoding RNAs in the initiation, progression, and metastasis of hepatocellular carcinoma. Noncoding RNA Res..

[24] Zhang H (2016). RNA helicase DEAD box protein 5 regulates Polycomb repressive complex 2/Hox transcript antisense intergenic RNA function in hepatitis B virus infection and hepatocarcinogenesis. Hepatology.

[25] Liu X (2018). Long non-coding RNA NEAT1-modulated abnormal lipolysis via ATGL drives hepatocellular carcinoma proliferation. Mol. Cancer.

[26] Hou Z (2017). HBx-related long non-coding RNA MALAT1 promotes cell metastasis via up-regulating LTBP3 in hepatocellular carcinoma. Am. J. Cancer Res.

[27] Bhan A, Mandal SS (2015). LncRNA HOTAIR: a master regulator of chromatin dynamics and cancer. Biochim Biophys. Acta.

[28] Chan JJ, Tay Y (2018). Noncoding RNA:RNA regulatory networks in cancer. Int. J. Mol. Sci..

[29] Qi X (2015). ceRNA in cancer: possible functions and clinical implications. J. Med. Genet..

[30] Tay Y, Rinn J, Pandolfi PP (2014). The multilayered complexity of ceRNA crosstalk and competition. Nature.

[31] Zhao Y (2019). Long noncoding RNA LINC02418 regulates MELK expression by acting as a ceRNA and may serve as a diagnostic marker for colorectal cancer. Cell Death Dis..

[32] Shao T (2019). The long noncoding RNA HOTAIR serves as a microRNA-34a-5p sponge to reduce nucleus pulposus cell apoptosis via a NOTCH1-mediated mechanism. Gene.

